# Saponin Biosynthesis in Pulses

**DOI:** 10.3390/plants11243505

**Published:** 2022-12-14

**Authors:** Bianyun Yu, Nii Patterson, L. Irina Zaharia

**Affiliations:** Aquatic and Crop Resource Development Research Center, National Research Council Canada, Saskatoon, SK S7N 0W9, Canada

**Keywords:** saponin biosynthesis, soyasaponin, pulse, oxidosqualene cyclases, cytochrome P450 monooxygenases, glycosyltransferases, regulation

## Abstract

Pulses are a group of leguminous crops that are harvested solely for their dry seeds. As the demand for plant-based proteins grows, pulses are becoming important food crops worldwide. In addition to being a rich source of nutrients, pulses also contain saponins that are traditionally considered anti-nutrients, and impart bitterness and astringency. Saponins are plant secondary metabolites with great structural and functional diversity. Given their diverse functional properties and biological activities, both undesirable and beneficial, saponins have received growing attention. It can be expected that redirecting metabolic fluxes to control the saponin levels and produce desired saponins would be an effective approach to improve the nutritional and sensory quality of the pulses. However, little effort has been made toward understanding saponin biosynthesis in pulses, and, thus there exist sizable knowledge gaps regarding its pathway and regulatory network. In this paper, we summarize the research progress made on saponin biosynthesis in pulses. Additionally, phylogenetic relationships of putative biosynthetic enzymes among multiple pulse species provide a glimpse of the evolutionary routes and functional diversification of saponin biosynthetic enzymes. The review will help us to advance our understanding of saponin biosynthesis and aid in the development of molecular and biotechnological tools for the systematic optimization of metabolic fluxes, in order to produce the desired saponins in pulses.

## 1. Introduction

Pulses are leguminous crops that are harvested strictly for their dry seeds, including peas, dry beans, lentils, chickpeas, faba beans, cow peas and pigeon peas. They are consumed worldwide, and have received growing attention due to both nutritional and environmental benefits. Pulses provide a rich source of protein, fiber, minerals and vitamins, as well as a healthy diet of low glycemic index that is also cholesterol-free and gluten-free. Pulses also contain an array of phytochemicals with various potential benefits for human health [1]. Saponins are widely present in the seeds of legumes, with variation in levels and composition across different species [2].

Saponins are a structurally diverse group of specialized plant terpenoids in many plant species, consisting of either steroidal or terpenoidal aglycones linked to oligosaccharide moieties [3,4]. In dicots, saponins feature a C30 triterpenoid hydrophobic core (sapogenin), whereas monocots predominantly contain sapogenin with a C27 steroidal structure [4]. Saponins usually occur as complex mixtures, and their composition and structure vary widely depending on the species, organ and tissue type, plant growth and development stage and environmental stimuli, both abiotic and biotic [5,6]. Based on hemolytic activity, saponins are generally classified into hemolytic (oleanates) and non-hemolytic (soyasapogenol) [7]. Saponins, although traditionally considered anti-nutrients, have received increasing recognition for their multiple potential health benefits.

It is generally believed that saponins contribute to the bitter and astringent taste of pulse-based food products [8]. Understanding the molecular mechanism underlying the biosynthesis and regulation of saponins is a prerequisite for modulating their content and composition toward crop improvement. In this review, we provide an overview of the biological functions of saponins and their distribution in pulse crops. Furthermore, this review primarily highlights the research progress made on the identification of the genes, enzymes and transcription factors in the saponin biosynthesis pathway in pulses. Owing to the availability of reference genome sequences, putative orthologs involved in saponin biosynthesis are identified in multiple pulse species. Phylogenetic relationships among these species are presented here, providing insight into the evolutionary routes and functional diversification of the saponin biosynthetic enzymes. This review will advance our knowledge of saponin biosynthesis, and will be informative for the development of genomic and molecular tools to manipulate saponin content and composition in pulse crops.

## 2. Biological Functions of Saponins

Saponins display a wide range of biological activities such as anti-microbial or anti-insect repellent activity, being produced as plant defense molecules in response to biotic threats, pathogens and herbivores [9]. Being a part of the antimicrobial defense system of plants, saponins change qualitatively and quantitatively in response to pathogen attacks [6]. The biotic stress from pea pathogens (*Aphanomyces euteiches*, or *Rhizoctonia solani* and *Fusarium oxysporum*) significantly increased the saponin content in grains of two pea (*Pisum sativum* L.) cultivars, Crécerelle and Firenza [10]. Additionally, saponins confer defensive measures against insects due to their bitter and astringent taste [11]. Given the growing concerns over the widespread use of synthetic pesticides, saponins are suggested to be environmentally friendly alternatives to conventional pesticides to provide crop protection [9]. In addition to being natural pesticides, saponins also enhance the abundance of the plant-beneficial microbes in the rhizosphere. Accumulating evidence has indicated that soyasaponins in legume root exudates promote the growth of the beneficial microbes that are associated with root nodulation and nitrogen fixation in the rhizosphere, although the composition of secreted soyasaponins is species-dependent and varies between the field and hydroponic culture conditions [9,12,13,14,15].

In the context of human health, saponins exhibit a wide spectrum of pharmacological properties, including anti-cholesterolemic, anti-cancer and hemolytic activities [2,4,9,16]. On the other hand, they can cause toxic effects to monogastric animals, and reduce forage palatability and digestibility in ruminants [17]. Apart from pharmacological applications, saponins are used in the detergent and cosmetic industries due to their foaming properties [18,19]. Saponins are also used as emulsifiers and foaming agents in the food industry [11]. Some saponins, such as glycyrrhizin, are used as sweeteners in the food industry [20], while others impart undesirable bitterness and astringency, reducing the palatability of pulse products [2,21,22].

## 3. Saponins in Pulse Crops

Soyasaponins, belonging to the oleanane triterpenoid group, are composed of soyasapogenol and oligosaccharide moieties, and are abundantly present in in the seeds of legumes such as soybean and pulses [2,9]. Four major groups of soyasaponins are distributed in legumes, namely soyasaponins A, B, E and DDMP (2,3-dihydro-2,5-dihydroxy-6-methyl-4H-pyran-4-one), which are categorized by their different substitutions at the C-21 and C-22 positions of the aglycone [23]. Group A and DDMP saponins are two major groups in soybeans [24]. Other than soybeans, soyasaponins A has been not reported in other legumes [9,22]. Group A saponins impart a bitter and astringent taste in soy-based food products, while group B (also known as soyasaponin I), group E and DDMP (also known as soyasaponin βg) soyasaponins are less bitter and confer health-promoting benefits for humans [21]. DDMP saponin differs from saponin B by the presence of a moiety at the C-22 position. Unstable DDMP saponin is converted to B and E saponins after hydrolysis during extraction and food processing [24,25,26]. DDMP saponins are the unique triterpenoids identified in legumes such as soybeans (*Glycine max*), peas (*Pisum sativum*) and chickpeas (*Cicer arietinum*) [24]. The content and composition of saponins in pulse seeds vary depending on the species and the genotypes within the species, and are also affected by the harvesting and storage conditions. DDMP saponin was more abundant than group B saponin in most of the 16 pea varieties studied, while DDMP saponin was the only saponin present in 2 out of 16 varieties [22]. Likewise, DDMP saponin was the predominant form, ranging from 80% to 92%, in mature seeds of six French dry pea cultivars and five landraces with different origins and/or end uses [23]. However, another study reported that group B saponin was predominant in another six different pea varieties [27]. One possible explanation for the disagreement between the aforementioned studies is the genetic backgrounds of the pea varieties used. The discrepancy might also result from the different extraction methods used and/or the degradation of DDMP saponin during extraction, leading to a proportional increase in group B saponin. Similarly, DDMP and group B saponins were the major groups detected in seeds of lentil (*Lens culinaris* Medik.), chickpea (*Cicer arietinum* L.), faba bean (*Vicia faba*) and dry bean (*Phaseolus vulgaris* L.) [28]. Both group B and DDMP saponins confer a bitter taste to the dry peas, with DDMP saponins significantly more bitter than the B saponins [22]. Seed size and testa color are associated with saponin content in lentils, with significantly higher saponin content identified in the varieties with larger seeds vs. smaller seeds, and in the varieties with beige or green testa compared to brown testa [29].

Processing and cooking reduce the total saponins, although to various degrees, in different pulse grains [30,31] and also cause the conversion of the higher molecular weight saponins to their lower mass analogues. However, soaking has relatively little effect on the saponin content [32], regardless of the pH of the soaking solution used [33]. These studies indicate that to a certain limited extent, processing and pre-treatment can reduce saponin content. However, a complicating issue is that processing also causes the loss of other nutrients. For instance, the canning process reduced the saponin content in chickpeas by up to 40%, but it consequently led to the loss of folate by up to 97% [34]. Further challenges come from the fact that processing consumes energy, and is less environment friendly. This highlights the need to develop the pulse varieties with desired saponin content and composition. Therefore, it is critically important to study the biosynthesis of saponins and develop genomic tools in order to modulate their composition and content, improve the sensory quality of pulse-based food products and impart health-promoting properties.

## 4. Saponin Biosynthesis

**Figure 1:**
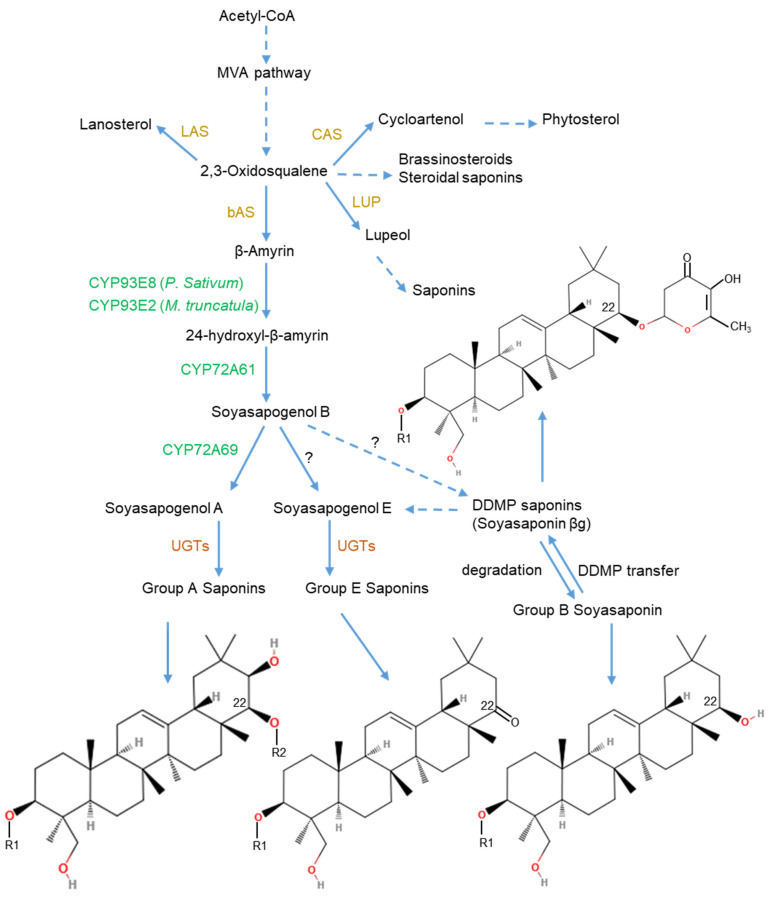
Soyasaponin biosynthesis pathway. MVA, mevalonate; bAS, β-amyrin synthase; CAS, cycloartenol synthase; LAS, lanosterol synthase; LUP, lupeol synthase; UGTs, uridine diphosphate-dependent (UDP) glycosyltransferases. Dashed lines indicate multiple steps involved. Compound structure was drawn using online tool molview, https://molview.org/ (accessed on 6 December 2022). References [9,11,35,39].

In this review, we will focus on the biosynthesis of triterpenoid saponins, since they are the class present in the pulses. Saponin biosynthesis involves sequential actions of three key classes of enzymes encoded by multi-gene families (Figure 1). The biosynthetic precursor of saponins is 2,3-oxidosqualene, derived from the cytosolic mevalonate (MVA) pathway [11]. Oxidosqualene cyclases (OSCs) construct various triterpenoid scaffolds via cyclization of a common precursor, 2,3-oxidosqualene, which is the first committed step in the biosynthesis of triterpenoid saponin. This step is also the first diversifying step, and a critical branching point for primary (phytosterol) and secondary triterpenoid (saponins) biosynthesis [35]. The triterpenoid skeletons formed after the cyclization step subsequently undergo various modifications by tailoring enzymes, including oxidation mediated by cytochrome P450 monooxygenases (P450s), to form the diverse triterpenoid aglycones and the glycosylation catalyzed by uridine diphosphate-dependent (UDP) glycosyltransferases (UGTs) [36,37]. Apart from UGTs, a recent study demonstrated that a cellulose synthase superfamily-derived glycosyltransferase (CSyGT) catalyzes 3-O-glucuronosylation of triterpenoid aglycones [38]. In addition, modifications by acyltransferases, malonyltransferases and methyltransferases are also involved in the biosynthesis of saponins, leading to a vast array of structural diversity.

Although significant progress has been made on saponin biosynthesis in the model legume *Medicago truncatula* and in soybeans, only a few genes have been cloned and characterized in pulse crops (Table 1). Many genes and enzymes remain uncharacterized in pulse crops, and their roles in this biosynthetic context have not been elucidated due to the complexity and functional diversification of these multi-gene families.

### 4.1. Oxidosqualene Cyclases (OSCs)

**Figure 2:**
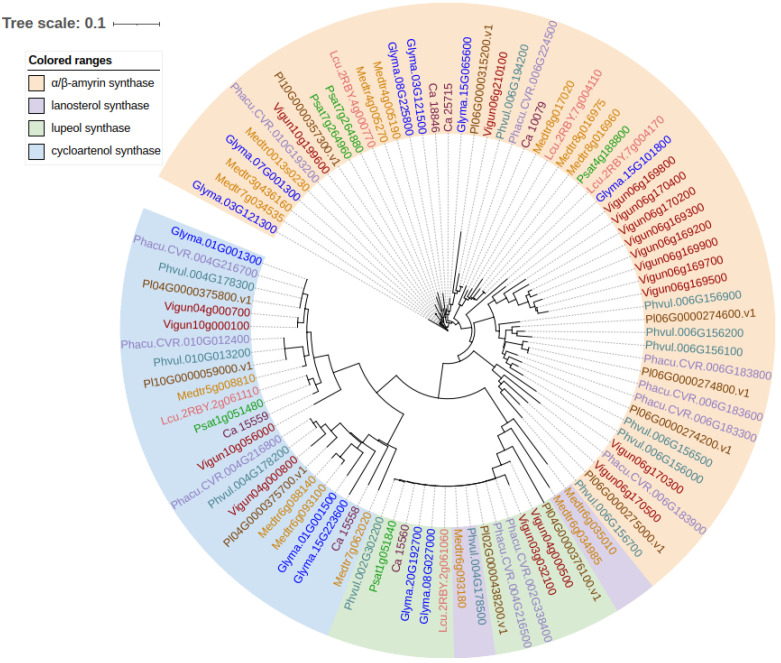
Neighbor-joining phylogenetic tree of oxidosqualene cyclases (OSCs) from pulse crop species. The amino acid sequences of pea (*Pisum Sativum*) OSCs were retrieved from the reference genome assembly of the cultivar Caméor (https://urgi.versailles.inra.fr/Species/Pisum/Pea-Genome-project, accessed on 6 December 2022). Pea OSCs were used for a blastp search against the proteomes of Medicago (*Medicago truncatula*, Mtruncatula_Mt4.0v1), common bean (*Phaseolus vulgaris* L., Pvulgaris_v2.1), chickpea (*Cicer arietinum* L., Carietinum_v1.0), soybean (*Glycine max*, Gmax_Wm82.a4.v1), cowpea (*Vigna unguiculata* L. Walp, Vunguiculata_v1.2), lima bean (*Phaseolus lunatus*, Plunatus_V1), tepary bean (*Phaseolus acutifolis* A. Gray, Pacutifolius_v1.0) and lentil (*Lens culinaris* Medik., Lculinaris_v1) on Phytozome 13 (https://phytozome-next.jgi.doe.gov/, accessed on 6 December 2022). Amino acid sequences were aligned using ClustalW with default parameters, and the neighbor-joining phylogenetic tree was constructed in the program DNASTAR Lasergene 17. Visualization of the tree was performed using online tool iTOL v6 (https://itol.embl.de/, accessed on 6 December 2022) [48].

OSCs are encoded by the multi-gene families to create various triterpene skeletons, including genes for β-amyrin synthase (bAS), cycloartenol synthase (CAS), lanosterol synthase (LAS), lupeol synthase (LUP) and several mixed-function OSCs that produce diverse products. These OSCs have specialized functions and make different major products [43]. A neighbor-joining phylogenetic tree was constructed with the protein sequences of five pea (*P. sativum*) OSC sequences reported by Vernoud et al. [23], together with 89 putative OSCs from the model legume *M. truncatula*, soybean (*G. max*) and pulse crops, including the common bean (*Phaseolus vulgaris* L.), tepary bean (*Phaseolus acutifolis* A. Gray), lima bean (*Phaseolus lunatus*), cowpea (*Vigna unguiculata* L. Walp), lentil (*Lens culinaris* Medik.) and chickpea (*C. arietinum*) (Figure 2). These OSCs are grouped into three major clusters, including α/β-amyrin synthase, cycloartenol synthase and lupeol synthase/lanosterol synthase, with about half of these genes belonging to the family of α/β-amyrin synthase. The number of OSC genes identified in these species varies from five (lentil) to eighteen (cowpea). Overall, multiple local tandem duplications are present for OSCs along the genome. CAS and LAS cyclize the 2,3-oxidosqualene to produce cycloartenol and lanosterol, respectively, the precursors for the biosynthesis of phytosterols. CAS is believed to be the ancestral OSC from which other OSCs are directly or indirectly derived by convergent evolution [44,45]. Expansion and functional diversification of OSCs are likely the result of multiple local tandem gene duplications and codon substitutions, with positive selection in response to environmental stresses, which allows them to acquire new functions [44]. Such tandem repeats of these gene families have been reported for the saponin biosynthesis in *M. truncatula* [46] and *Barbarea vulgaris* [47], suggesting the likelihood that such gene organization facilitates the creation of multiple end products with a limited number of genes [47].

A few OSCs have been isolated and functionally characterized in peas. Characterization of two ethyl methanesulfonate (EMS)-induced pea mutants, *bas1,* in two different genetic backgrounds led to the identification of the *PsBAS1* gene (*Psat7g264880*) encoding a β-amyrin synthase that was expressed in several tissues, including the developing seeds [23]. The expression of *PsBAS1* was much higher in the seed embryo than the seed coat, which coincided with the accumulation patterns of both DDMP and type B saponins. The expression of *PsBAS1* was first detected at 19 days after pollination (dap) and increased as the seed matured with the peak at 29 dap [23]. The gene expression pattern of *PsBAS1* throughout the pea seed development was in agreement with the accumulation pattern of saponin content, which was strongly increased in the late stage, indicating a key role of *PsBAS1* in the biosynthesis of saponin [23]. Two triterpene synthase cDNAs were cloned from immature seeds of *P. sativum*, with one encoding β-amyrin synthase (PSY) [40] that was identical to *Psat7g264880,* and the other encoding mixed amyrin synthase (PSX) to produce both α- and β-amyrin [40], which had a 98% identity to *Psat4g188800* at the amino acid level. A cDNA encoding cycloartenol synthase was isolated from pea seedlings [41], with 99% identity to *Psat1g051480*. To our knowledge, OSCs have not been characterized in other pulse crops. Functional characterization of these OSCs is necessary to advance our knowledge of saponin biosynthesis and to facilitate the development of molecular tools to modify saponin content in pulse crops.

### 4.2. Cytochrome P450 Monooxygenases (P450s)

The second diversifying step for triterpenoid biosynthesis occurs when P450s catalyze the site-specific oxidation of the cyclized skeleton, primarily through the introduction of hydroxyl, carboxyl or epoxy groups [43]. A large number of triterpenoid biosynthesis-related P450s have been identified in different species, including the subfamilies CYP93 and CYP72 [35,42,49]. The CYP93E family is a member of the functionally diverse CYP71 clan, and is made up of five subfamilies (A–E), with subfamily E involved in triterpenoid saponin metabolism [49]. CYP93E orthologs are common in the *Leguminosae* family, and have yet to be found outside the *Leguminosae*. Unlike CYP93E, the CYP72A subfamily is distributed across the entire plant kingdom [50]. The specific function and catalytic efficiency of each of these P450s vary.

**Figure 3:**
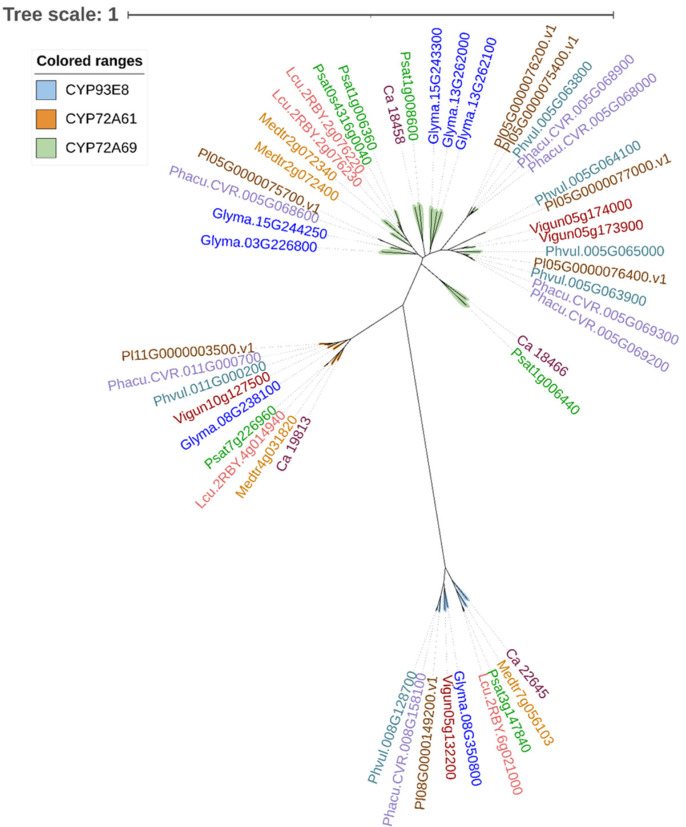
Neighbor-joining phylogenetic tree of cytochrome P450 monooxygenases (P450s) from pulse crop species. The amino acid sequences of pea CYP93E8, Medicago CYP72A61 and soybean CYP72A69 were retrieved from GenBank (accession AIN25420.1, ABC59088.1 and BAW35014.1, respectively) and were used for a blastp search against the proteomes of Medicago (*Medicago truncatula*, Mtruncatula_Mt4.0v1), common bean (*Phaseolus vulgaris* L., Pvulgaris_v2.1), chickpea (*Cicer arietinum* L., Carietinum_v1.0), soybean (*Glycine max*, Gmax_Wm82.a4.v1), cowpea (*Vigna unguiculata* L. Walp, Vunguiculata_v1.2), lima bean (*Phaseolus lunatus*, Plunatus_V1), tepary bean (*Phaseolus acutifolis* A. Gray, Pacutifolius_v1.0) and lentil (*Lens culinaris* Medik., Lculinaris_v1) on Phytozome 13 (https://phytozome-next.jgi.doe.gov/, accessed on 6 December 2022) and pea (*Pisum sativum*) cultivar Caméor (https://urgi.versailles.inra.fr/Species/Pisum/Pea-Genome-project, accessed on 6 December 2022). Amino acid sequences were aligned using ClustalW with default parameters, and the neighbor-joining phylogenetic tree was constructed in the program DNASTAR Lasergene 17. Visualization of the tree was performed using the online tool iTOL v6 (https://itol.embl.de/, accessed on 6 December 2022) [48].

Phylogenetic analysis of putative P450s involved in soyasaponin biosynthesis distinguishes three distinctive clades (Figure 3). Only a single member is present in each species for both the CYP93E and CYP72A61 families. In contrast, multiple members are identified in each species for the CYP72A69 family, and most are present in tandem repeats along the genome. In each P450 family, orthologs from *P. sativum*, *M. truncatula*, *L. culinaris* Medik. and *C. arietinum* L. are clustered together, suggesting that these species likely share a common ancestry. The CYP93E subfamily catalyzes the C-24 oxidation of the triterpene backbone (β-Amyrin) to produce 24-OH-β-amyrin during the biosynthesis of triterpenoid saponins [42]. Different catalytic efficiencies between CYP93E2 (*M. truncatula*) and CYP93E8 (*P. sativum*) were demonstrated in a heterologous host (*Saccharomyces cerevisiae*) [42]. The CYP72A subfamily, with over 266 members, is widely present in plants. The members of the CYP72A subfamily catalyze different reactions, leading to the production of diverse triterpenoids in legumes. Both soybean CYP72A61 and the *M. truncatula* ortholog CYP72A61v2 transform 24-OH-β-amyrin into the major soyasaponin aglycone, soyasapogenol B, by catalyzing the hydroxylation at the C-22 position [51]. Soyasapogenol B can be further oxidized at the C-21 position to produce soyasapogenol A (Figure 1) by CYP72A69 in soybean (*G. max*) [21,52,53]. Soyasapogenol A subsequently undergoes glycosylation, catalyzed by UGTs. The presence of the C-21 hydroxyl group is considered to be highly specific to soyasaponin A [53]. A loss-of-function mutation in *Glyma15g39090* encoding the cytochrome P450 enzyme, CYP72A69, led to metabolic switching from undesirable group A saponins to beneficial DDMP saponins in soybean [21]. The identification of such a mutant provides a new avenue for developing soybean cultivars with improved sensory quality and increased consumer acceptance of soy-based food products.

### 4.3. Uridine Diphosphate-Dependent (UDP) Glycosyltransferases (UGTs)

Glycosylation is the last modification step in the biosynthesis of saponins. This sugar conjugation step is important for the biological activities and chemical properties of saponins by regulating triterpenoid aglycones, including increasing their stability, water solubility and cell compartmentalization [54]. The glycosylation confers enormous structural and functional diversity to saponins, with variations in the number, the position and the composition of sugar moieties [24,55]. An array of UGTs, including the UGT71, UGT73, UGT74 and UGT91 families, are involved in the glycosylation of saponins, which transfer UDP-activated sugars to the acceptor [24,43,56,57,58,59,60]. In terms of the acceptor, UGTs can be either highly selective or catalyze different classes of compounds [61]. For instance, UGT71G1 (*M. truncatula*) can glycosylate certain isoflavones and the flavonol quercetin, in addition to triterpenoids [16]. Most of the UGTs identified in saponin biosynthesis catalyze sugar transfer at the positions C3 and C28 [37]. In *M. truncatula*, UGT73K1 was demonstrated to have specificity for hederagenin and soyasapogenols B and E, while UGT71G1 has specificity for medicagenic acid [16]. In soybean, soyasaponin I is biosynthesized from soyasapogenol B by successive sugar transfer reactions, catalyzed by UGT73P2. This adds a galactose to the C-3-linked glucuronic acid of soyasapogenol B, and a second enzyme, UGT91H4, subsequently adds a rhamnose to the galactose moiety [62]. The variation of UGT73F4 alleles was shown to be responsible for the differential accumulations of soyasaponin Aa and Ab between two soybean cultivars [63].

**Figure 4:**
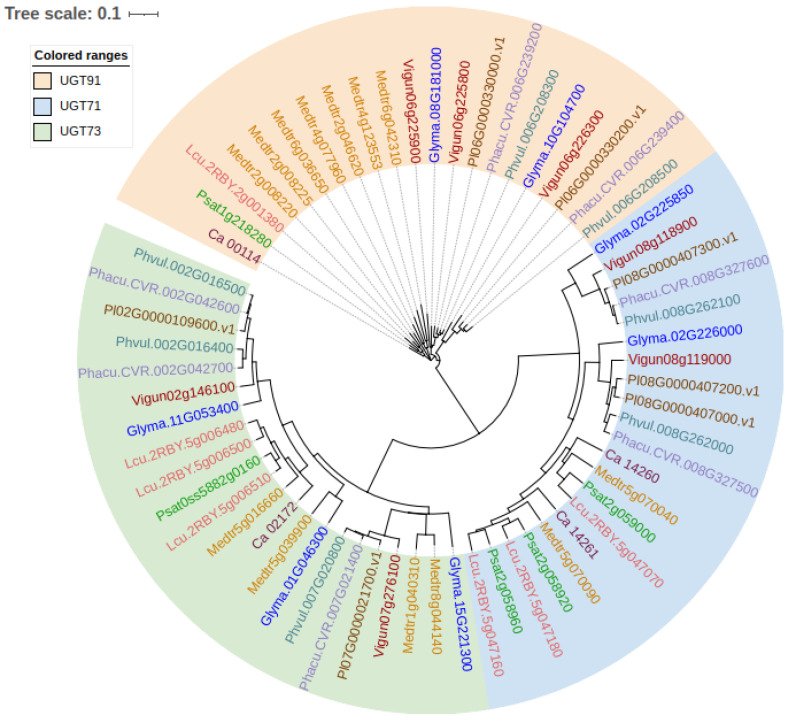
Neighbor-joining phylogenetic tree of uridine diphosphate-dependent (UDP) glycosyltransferases (UGTs) orthologues from pulse crop species. The amino acid sequences of Medicago UGT71G1, soybean UGT73P2 and soybean UGT91H4 were retrieved from GenBank (accession AAW56092, BAI99584 and BAI99585, respectively) and were used for a blastp search against the proteomes of Medicago (*Medicago truncatula*, Mtruncatula_Mt4.0v1), common bean (*Phaseolus vulgaris* L., Pvulgaris_v2.1), chickpea (*Cicer arietinum* L., Carietinum_v1.0), soybean (*Glycine max*, Gmax_Wm82.a4.v1), cowpea (*Vigna unguiculata* L. Walp, Vunguiculata_v1.2), lima bean (*Phaseolus lunatus*, Plunatus_V1), tepary bean (*Phaseolus acutifolis* A. Gray, Pacutifolius_v1.0) and lentil (*Lens culinaris* Medik., Lculinaris_v1) on Phytozome 13 (https://phytozome-next.jgi.doe.gov/, accessed on 6 December 2022) and pea (*Pisum sativum*) cultivar Caméor (https://urgi.versailles.inra.fr/Species/Pisum/Pea-Genome-project, accessed on 6 December 2022). Amino acid sequences were aligned using ClustalW with default parameters, and the neighbor-joining phylogenetic tree was constructed in the program DNASTAR Lasergene 17. Visualization of the tree was performed using the online tool iTOL **v6** (https://itol.embl.de/, accessed on 6 December 2022) [48].

A neighbor-joining phylogenetic tree for the subfamilies of putative UGT71, UGT73 and UGT91 orthologs in pulse crops (Figure 4) shows that the UGT71 and UGT73 families are more similar compared to the UGT91 family. Similar to P450s, the orthologs from *P. sativum*, *M. truncatula*, *L. culinaris* Medik. and *C. arietinum* L. are clustered together. Multiple members are present in each of the subfamilies. However, further experimental studies are required to confirm whether they are involved in saponin biosynthesis, as well as what their functional mechanism is. It is not completely clear which enzymes are involved in the biosynthesis of DDMP soyasaponins. However, a member of UGT73B4, encoded by the gene *Glyma.16G033700* in soybeans, has been suggested to function as a DDMP transferase, catalyzing the production of DDMP saponins from group B saponins [24] and/or another suggested precursor, soyasapogenol B [36].

## 5. Regulation of Saponin Biosynthesis

The biosynthesis and accumulation of saponin are subjected to the transcriptional, developmental and spatiotemporal modulation [35]. Prior studies have highlighted the key roles of the phytohormones jasmonate (JA) and methyl jasmonate (MeJA) in regulating the expression of saponin biosynthesis genes [9,64,65,66,67]. Transcription factors (TFs) in the families WRKY, APETALA2/Ethylene Response Factor (AP2/ERF) and Basic Helix-Loop-Helix (bHLH) positively regulate triterpenoid saponin biosynthesis induced by MeJA signaling [4,9,68]. For instance, a MeJA-inducible member of the WRKY TF gene family acts as a positive regulator of triterpene ginsenoside biosynthesis in American ginseng (*Panax quinquefolius* L) [69]. In the model legume *M. truncatula*, two JA-inducible bHLH TFs, TRITERPENE SAPONIN BIOSYNTHESIS ACTIVATING REGULATOR1 (TSAR1) and TSAR2 positively regulate non-hemolytic and hemolytic triterpenoid saponin biosynthesis, respectively [67]. A seed-specific bHLH transcription factor, TRITERPENE SAPONIN ACTIVATION REGULATOR3 (TSAR3), encoded by *Medtr2g104650,* regulates the expression of CYP88A13 in the developing seeds of *M. truncatula*, a cytochrome P450 that catalyzes the final oxidation step of the hemolytic saponin biosynthesis branch [11]. Although it seems that TSAR1 is the naturally preferred regulator of soyasaponin biosynthesis, available experimental evidence supports that TSAR2 and TSAR3 also participate in the production of soyasaponin [11]. Compared to the positive regulators, fewer negative regulators have been identified for saponin biosynthesis. The basic leucine zipper (bZIP) TFs, bZIP17 and bZIP60, downregulate JA-dependent triterpenoid saponin synthesis in *M. truncatula* by suppressing the activities of TSAR1 and TSAR2 [70].

In addition to TFs, microRNAs (miRNAs) have been reported to be involved in terpenoid biosynthesis and accumulation in plants [71]. The miRNAs, which are small (21–24 nucleotides) non-coding molecules, regulate plant development, environmental responses of the plants and secondary metabolism. A recent study suggested that miR2093-5p, miR4414b, miR5037a, miR829-3p.1 and miR838-3p play crucial roles in the regulation of triterpenoid saponin in *Gleditsia sinensis* Lam. (Leguminosae) [72]. However, no study has been reported on the identification and characterization of the miRNAs involved in saponin biosynthesis in pulse crops.

The dynamic accumulation of secondary metabolites, including saponins, in response to environmental stimuli is an adaptive strategy of the plants which allows them to survive under adverse environmental conditions. Abiotic environmental factors, such as temperature and precipitation, affect the accumulation patterns of saponins, including their content and composition [6]. Increased accumulation of saponin was observed in the leaves of *Quillaja brasiliensis* when exposed to abiotic elicitors such as salicylic acid, jasmonic acid, UV light and mechanical damage [73]. Furthermore, saponins might play a key role in plant salinity tolerance [74]. With inevitable climate changes in the frequency and severity of drought, further research is necessary to unravel the molecular mechanism of saponin fluctuation in response to environment changes, leading to tolerance toward stressors.

## 6. Future Perspectives

Triterpenoid saponin biosynthesis in pulses is poorly understood, and many pathway genes remain uncharacterized. Rapid advances in next generation sequencing technology promote the increasing availability of genome and pan-genome sequences of multiple crop species, which no doubt accelerates the discovery of new genes/alleles involved in saponin biosynthesis. Integrated applications of the genome, transcriptome, proteome and metabolome are opening up unprecedented opportunities for functional genomics to elucidate the molecular mechanism underlying triterpenoid saponin metabolism in pulse crops. In conjunction with exploiting natural gene variants in diverse germplasms, genetic engineering with CRISPR and/or molecular breeding for targeted production of saponins will facilitate the development of the varieties with balanced benefits to plant health, nutritional quality for human and animal consumption, and palatability for pulse food products.

## Figures and Tables

**Table 1:** Saponin biosynthesis genes identified in pulse crops.

Gene Name	Gene ID/GenBank Accession Number	Gene Function	Crop	Reference
Oxidosqualene cyclases (OSCs)				
β-amyrin synthase1	*Psat7g264880*	producing β-amyrin	Pea (*Pisum sativum*)	[23]
β-amyrin synthase	AB034802	producing β-amyrin	Pea (*Pisum sativum*)	[40]
α- and β-mixed amyrin synthase	AB034803	producing both α- and β-amyrin	Pea (*Pisum sativum*)	[40]
cycloartenol synthase	D89619		Pea (*Pisum sativum*)	[41]
Cytochrome P450-dependent monooxygenases (P450s)				
CYP93E5	KF906536	C-24 oxidation in triterpenoid saponin	Chickpea (*Cicer arietinum* L.)	[42]
CYP93E7	KF906538	C-24 oxidation in triterpenoid saponin	Lentil (*Lens culinaris* Medik.)	[42]
CYP93E8	KF906539	C-24 oxidation in triterpenoid saponin	Pea (*Pisum sativum*)	[42]
CYP93E9	KF906540	C-24 oxidation in triterpenoid saponin	Dry bean (*Phaseolus vulgaris* L.)	[42]

## Data Availability

Not applicable.
